# Early Probiotic Supplementation and the Risk of Celiac Disease in Children at Genetic Risk

**DOI:** 10.3390/nu11081790

**Published:** 2019-08-02

**Authors:** Ulla Uusitalo, Carin Andren Aronsson, Xiang Liu, Kalle Kurppa, Jimin Yang, Edwin Liu, Jennifer Skidmore, Christiane Winkler, Marian J. Rewers, William A. Hagopian, Jin-Xiong She, Jorma Toppari, Anette-G. Ziegler, Beena Akolkar, Jill M. Norris, Suvi M. Virtanen, Jeffrey P. Krischer, Daniel Agardh, on behalf of the TEDDY Study Group

**Affiliations:** 1 Health Informatics Institute, Department of Pediatrics, Morsani College of Medicine, University of South Florida, Tampa, FL 33612, USA; 2 The Diabetes and Celiac Disease Unit, Department of Clinical Sciences, Lund University, 214 28 Malmö, Sweden; 3 Tampere Center for Child Health Research, University of Tampere, 33014 Tampere, Finland; 4 The University Consortium of Seinäjoki, 60320 Seinäjoki, Finland; 5 Digestive Health Institute, Children’s Hospital Colorado, University of Colorado Denver, Aurora, CO 80045, USA; 6 Pacific Northwest Research Institute, Seattle, WA 98122, USA; 7 Institute of Diabetes Research, Helmholtz Zentrum München, 85764 Munich-Neuherberg, Germany; 8 Forschergruppe Diabetes, Klinikum rechts der Isar, Technische Universität München, 80804 Munich, Germany; 9 Forschergruppe Diabetes e.V., Helmholtz Zentrum München, 85764 Munich-Neuherberg, Germany; 10 Barbara Davis Center for Childhood Diabetes, University of Colorado School of Medicine, Aurora, CO 80045, USA; 11 Medical College of Georgia, Augusta University, Augusta, GA 30912, USA; 12 Research Centre for Integrative Physiology and Pharmacology, Institute of Biomedicine, University of Turku, 20014 Turku, Finland; 13 Department of Pediatrics, Turku University Hospital, 20521 Turku, Finland; 14 NIDDK, National Institute of Health, Bethesda, MD 20892, USA; 15 Department of Epidemiology, Colorado School of Public Health, University of Colorado, Aurora, CO 80045, USA; 16 Unit of Nutrition, National Institute for Health and Welfare, 00271 Helsinki, Finland; 17 Tampere University Hospital, and the Science Center of Pirkanmaa Hospital District, 33520 Tampere, Finland

**Keywords:** probiotics, dietary supplements, infant formula, celiac disease autoimmunity, celiac disease

## Abstract

Probiotics are linked to positive regulatory effects on the immune system. The aim of the study was to examine the association between the exposure of probiotics via dietary supplements or via infant formula by the age of 1 year and the development of celiac disease autoimmunity (CDA) and celiac disease among a cohort of 6520 genetically susceptible children. Use of probiotics during the first year of life was reported by 1460 children. Time-to-event analysis was used to examine the associations. Overall exposure of probiotics during the first year of life was not associated with either CDA (*n* = 1212) (HR 1.15; 95%CI 0.99, 1.35; *p* = 0.07) or celiac disease (*n* = 455) (HR 1.11; 95%CI 0.86, 1.43; *p* = 0.43) when adjusting for known risk factors. Intake of probiotic dietary supplements, however, was associated with a slightly increased risk of CDA (HR 1.18; 95%CI 1.00, 1.40; *p* = 0.043) compared to children who did not get probiotics. It was concluded that the overall exposure of probiotics during the first year of life was not associated with CDA or celiac disease in children at genetic risk.

## 1. Introduction

Probiotics have been defined as live organisms which confer a health benefit to the host when administered in adequate amounts [1]. There is a long history of safe use of selected microbes in foods, but the vulnerability of certain immunocompromised populations should be taken into consideration before probiotic supplementation [2]. Administration of probiotics to healthy infants is considered to be safe [3,4] and is assumed to have a positive effect on the regulation of the immune system. Probiotic supplementation has long been used for its preventive effects, e.g., on atopic eczema [5,6] and to improve and maintain gastrointestinal health [7]. Whether probiotics are beneficial against colic pain and decrease the related crying time in young infants remains a controversial topic [8,9,10,11]. Nevertheless, supplementation of infant diets with probiotics has become more common worldwide during the recent years. For instance, a considerable increase in probiotic supplement use has been observed in Sweden since 2004 [12,13], while infants in Finland have been commonly given probiotics since the late 1990s [14]. Despite the increase in probiotic use, their mechanisms of action have not entirely been established. Furthermore, it has been suggested that dead bacteria and their components can also exhibit probiotic properties, in addition to probiotics and probiotic metabolites [15].

Celiac disease is a chronic autoimmune disease of the small bowel, characterized by villous atrophy and inflammation of the intestinal mucosa [16]. Early infant feeding, with emphasis on gluten intake, influences risk [17]. While gluten is the necessary antigen for celiac disease to develop, there is some evidence that various environmental exposures such as repeated gastrointestinal infections may also trigger the disease [18,19,20], either on their own or in interaction with gluten exposure [21]. Certain changes in gut microbiota composition, as well as the development and maturity level of the gut microbiota, have been linked to celiac disease [21,22]. Some previous studies have also shown that probiotics are beneficial for children who suffer from this autoimmune disorder [23,24]. 

We previously demonstrated that early exposure of probiotics may decrease the risk of type 1 diabetes (T1D) related autoimmunity among at-risk children in the ongoing prospective, The Environmental Determinants of the Diabetes in the Young (TEDDY) birth cohort study [12] which is a multi-national longitudinal observational study with the goal of identifying environmental factors associated with T1D and celiac disease. To date there is no prospective study showing that probiotics may prevent celiac disease. The aim of this study was to examine whether the timing of initial probiotic exposure during the first year of life is associated with the risk of celiac disease autoimmunity (CDA) and celiac disease in the TEDDY study.

## 2. Materials and Methods

### 2.1. Study Population

The TEDDY study involves 6 clinical research centers located in Colorado, Georgia, and Washington in the U.S. and in Finland, Germany, and Sweden in Europe. All sites follow the same study protocol including scheduled visits every 3rd month until the age of 4 years and every 6th month thereafter until 15 years of age [25]. Between September 2004 and February 2010, the TEDDY study screened 424,788 newborns infants of whom 21,589 infants fulfilled the inclusion criteria based on the Human Leukocyte Antigen (HLA) genotyping (Supplemental Table S1). Among those eligible children, 8676 children were enrolled in the prospective cohort study. For this study we only included children with selected HLA genotypes: DR3/3, DR3/4, DR4/4, and DR4/8 who had been screened for tissue transglutaminase autoantibodies (tTGA) (*n* = 6520) (Figure S1). As of July 2017, 6520 children had been followed to a median (interquartile range (IQR)) age of 8.7 (7.4–10.2) years and included for this study.

For all study participants, separate written informed consent for genetic screening and participation in the follow-up study were obtained from a parent or primary caregiver before they participated in the study. The study was conducted in accordance with the Declaration of Helsinki, and the protocol was approved by local Ethics Committees and Institutional Review Boards and monitored by an External Evaluation Committee formed by the National Institutes of Health.

### 2.2. Screening for Celiac Disease Related Autoimmunity (CDA) and Celiac Disease

Annual screening for celiac disease starts with tTGA at 2 years of age, as previously described [26]. Children who are tTGA positive are re-tested after 3–6 months and defined as having CDA if persistently tTGA positive in two consecutive samples. In addition, children who tested tTGA positive had their serum samples retrospectively analyzed. Samples from as early as 3 months of age were available in order to determine the closest time-point of seroconversion. Caregivers to children with CDA were referred to their health care provider for further evaluation of celiac disease. For the purpose of this study, celiac disease was defined as biopsy proven (i.e., an intestinal biopsy showing a Marsh score ≥ 2) or having a persistently tTGA level of ≥100 units if a biopsy was not performed [27]. 

### 2.3. Information on Characteristics, Diet and Health of the Study Population

Information about basic demographic characteristics of the mother and her newborn baby was received from the infant screening form. A questionnaire was mailed to the home of the mother prior to the first clinic visit (3 to 4.5 months postpartum). This questionnaire contained questions regarding illnesses during pregnancy, mother’s use of medications and dietary supplements, smoking status, and maternal body mass index (BMI) before pregnancy. After enrollment, families received a questionnaire on the mode of delivery and the child’s early diet, including use of probiotics between 0 to 3 months of age. Information about the mother’s education and the birth order of the child was received from the primary caretaker questionnaire at the 9-month clinic visit. Parents were advised to consistently maintain a diary after the first clinic visit in order to collect information on child illnesses and diet. The child’s age at the start and end of probiotic supplementation and/or infant formula, as well as of each type of formula, were recorded. Probiotic exposure was defined as the timing of first probiotic introduction of either via dietary supplement or infant formula. Species of probiotics in supplements and infant formulas were examined based on the composition of the self- reported brand name products. The majority of the probiotic bacteria in dietary supplements and infant formulas taken by the TEDDY children consisted of *Lactobacillus reuteri* and *Lactobacillus rhamnosus*, although 17% of the families were not able to identify the brand name of the probiotics they used. The latter was more likely to occur during the first 3 months of age, when the information on probiotic intake was recalled retrospectively. Study nurses reviewed the questionnaires and diaries during the family’s clinic visit or over the phone every 3 months to minimize missing or inaccurate information. 

### 2.4. Statistical Methods 

Time-to-event analysis with Cox proportional hazards (PH) modeling was performed to examine the overall probiotic exposure and timing in the first year of life in relation to the risk of CDA and celiac disease. The magnitudes of the associations were described by hazard ratios (HR) with 95% confidence intervals (CI). The Cox PH models were adjusted for known risk factors for celiac disease including sex, HLA genotype, family history of celiac disease (first-degree relative (FDR) with celiac disease vs. not), and country (as strata). In addition, Cox PH models were adjusted for potential confounders associated with both probiotics use and CDA or celiac disease, including birth year, mode of delivery, mother’s education, duration of exclusive breastfeeding, and child’s diarrhea status in the first 3 months.

Probiotic exposure in the first year of life was incorporated into the Cox PH model in two ways: (a) probiotic exposure was categorized as binary (yes vs. no): probiotics users vs. non-users, and (b) source of probiotic exposure was categorized into three groups: dietary supplements, infant formula, or none. Among probiotics users, the age at first probiotic exposure was examined as a continuous variable in the Cox PH model. Analyses were carried out using the Statistical Analysis System software (version 9.4; SAS Institute, Cary, NC, USA).

## 3. Results

### 3.1. Study Population

During follow-up, 1212 (18.6%) children were identified as having CDA at a median (IQR) age of 3.3 (2.2–5.0) years (range 0.9–11.5 years), while 455 (7.0%) children were diagnosed with celiac disease at a median (IQR) age of 4.3 (3.2–6.2) years (range 1.2–12.5 years). The characteristics of the children by the status of CDA or celiac disease are presented in Table 1.

### 3.2. Probiotic Use

A total of 1460 children were exposed to probiotics via dietary supplements or infant formula during the first year of life. The characteristics of the children by the source of probiotic exposure in the first year of life are presented in Table 2. The participants’ characteristics that were positively associated with probiotics use during the first year of life were country (*p* < 0.001), later birth year (*p* < 0.001), mode of delivery (other than Cesarean section) (*p* = 0.012), being the first born child (*p* < 0.001), older maternal age (*p* = 0.001), higher maternal education (*p* < 0.001), not smoking during pregnancy (*p* = 0.005), maternal probiotic use during pregnancy (*p* < 0.001), shorter duration of exclusive breastfeeding (*p* < 0.001), antibiotic use (*p* < 0.001), diarrhea during the first 3 months (*p* < 0.001), gastrointestinal infections (*p* < 0.001), and lower incidence of common cold during the first 3 months (*p* = 0.006) (Table 2). There was a considerable increase in probiotics use by birth year in Sweden (Figure S2) when compared to other countries where there was not as much difference in the probiotics use across the study years.

### 3.3. Risk of CDA and Celiac Disease

There was no difference in the risk of CDA (HR 1.15; 95%CI 0.99, 1.35; *p* = 0.07) or celiac disease (HR 1.11; 95%CI 0.86, 1.43; *p* = 0.43) between probiotics users and non-users during the first year of life when the models were adjusted for potential confounders: country, sex, HLA-genotype, FDR with celiac disease, birth year, mode of delivery, mother’s education, duration of exclusive breastfeeding, and child’s diarrhea during the first 3 months (Table 3). However, probiotic exposure from dietary supplements alone when compared to no exposure was associated with a slightly increased risk of CDA when adjusted for the potential confounders (HR 1.18; 95%CI 1.01, 1.40; *p* = 0.043) (Table 3). 

**Figure 1:**
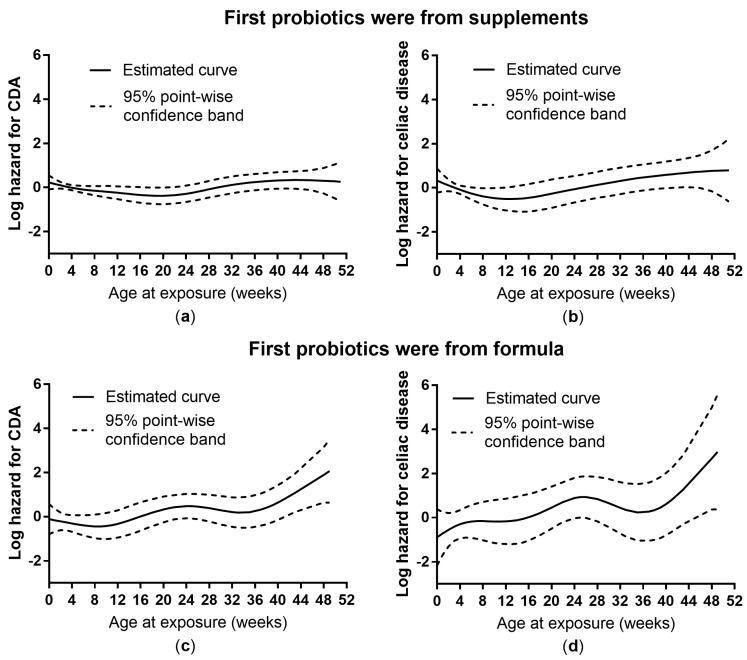
The estimated effects of age at probiotic exposure (by source of probiotics) on the log hazards of celiac disease autoimmunity (CDA, *n* = 281) (**a**,**c**; nonlinearity: *p* = 0.054 and *p* = 0.16, respectively) and celiac disease (*n* = 99) (**b**,**d**; nonlinearity: *p* = 0.16 and *p* = 0.20, respectively) from time-to-event analysis with smoothing splines on 1460 subjects who were exposed to probiotics during the first year of life.

No association was found between the age of the child at the time of initial probiotic exposure and the risk of the outcomes when adjusting for all the potential confounders (Table 3). However, a time-to-event analysis with smoothing splines [28,29] showed a slightly increased subsequent risk of celiac disease when a probiotic dietary supplement was introduced during the first weeks of life (Figure 1).

## 4. Discussion

The present study showed no protective association between overall probiotics use during first year of life and the risks of CDA or celiac disease in children at increased risk of T1D and celiac disease. In fact, probiotic exposure from dietary supplements during the first weeks of life was associated with a small increase in the risk of celiac disease. This finding is in contrast to a recent randomized double-blinded placebo-controlled study in Sweden in which synergistic effects of probiotics on the peripheral autoimmune response were observed in genetically predisposed children with CDA who were receiving two strains of *Lactobacillus*, as compared to placebo [30]. However, to our knowledge no other study has investigated the association between probiotic use and celiac disease in children in a prospective multicenter study like TEDDY. 

Probiotic use in the first year of life was positively linked to various infections and antibiotic use in children. This finding could be interpreted as infectious episodes potentially acting as a confounder when studying the association between probiotic exposure and the outcomes. However, we did not find any association between infections or antibiotic use and the risk of CDA or celiac disease in this study. Probiotics use was associated with shorter exclusive breastfeeding, which could have been related to earlier introduction of gluten-containing cereals. However, there is no current evidence that early introduction of gluten could be linked to the risk of celiac disease risk [31,32]. Moreover, the association between breastfeeding duration and the risk of celiac disease still remains inconclusive [33].

Administration of antibiotics was not associated with CDA or celiac disease in this study, which is in line with an earlier report from the TEDDY Study [20] in which antibiotic use between 3 months and 4 years of age was examined in relation to CDA and celiac disease, adjusting e.g. for probiotic use during the first 3 months. However, two recent studies suggest that taking antibiotics at an early age is associated with celiac disease [34,35], although neither of these two studies took potential early probiotic exposure into account. 

Colic usually appears during the first 2–3 weeks of life and a common practice, especially in Sweden, involves recommending probiotic supplement drops to ease the abdominal discomfort caused by colic [36]. Unfortunately, colicky events were not recorded in this study and their role as a potential confounder could not be further evaluated. Nevertheless, the etiology of both colic [37,38] as well as celiac disease [39] has been associated with dysbiosis in gut microbiota, and both are also associated with similar health conditions [40,41]. 

We also speculated whether use of probiotics after the first year of life could be linked to the risk of CDA or celiac disease. Because yoghurt and other fermented milk products are frequently given to young children especially after the first year of age, probiotics in the form of dietary supplements or infant formula could seldom be counted as the first exposure at that age. Food sources of probiotics (e.g., *Lactobacillus* species via fermented milk and vegetable products or fortified foods), would then become more frequent in infant diet, minimizing the importance of dietary supplements as the first probiotic exposure. Furthermore, given suggestions that gut microbiota is an important part of the causal pathway of celiac disease, we have to take into consideration that gut microbiota has already been shaped by solid foods after 1 year of age [42] and the long-term modification of gut microbiota by introduction of probiotic supplements at that time would probably not be feasible.

The strength of this study is the prospective design including subjects from multiple international clinical centers, as well as using standardized and validated methods in data collection across the study centers. The mechanistic actions of probiotic bacteria could be dependent on species and doses of probiotics, for example. Since this information was not available for this study, it suffers from limitations despite its large size and prospective design. However, there is currently no definite consensus by which specific bacteria could be associated with celiac disease [22,43]. The dose of probiotics could not be studied because of the lack of information on the manufacturing processes and storage conditions [44] of the large variety of probiotic supplements and infant formulas that were used by the study participants. There was also limited information about early life events and their timing due to the retrospective collection of self-reported data at the age of 3 months.

## 5. Conclusions

This study demonstrated that the overall supplementation of probiotics in infancy was not associated with celiac disease. However, the finding that first probiotic exposure from dietary supplements during the first weeks of life was associated with the increased risk of celiac disease warrants further investigation.

## Figures and Tables

**Table 1:** Characteristics of the study participants by status of celiac disease autoimmunity (CDA) and of celiac disease.

	Developed CDA (*n* = 1212)	Did not Develop CDA (*n* = 5308)	Developed Celiac Disease (*n* = 455)	Did not Develop Celiac Disease (*n* = 6065)
Country				
-US	437 (36.1)	2195 (41.4)	135 (29.7)	2497 (41.2)
-Finland	257 (21.2)	1227 (23.1)	85 (18.7)	1399 (23.1)
-Germany	58 (4.8)	289 (5.4)	17 (3.7)	330 (5.4)
-Sweden	460 (37.9)	1597 (30.1)	218 (47.9)	1839 (30.3)
Family member with celiac disease	123 (10.1)	136 (2.6)	76 (16.7)	183 (3.0)
Sex, male	517 (42.7)	2815 (53.0)	167 (36.7)	3165 (52.2)
HLA DR-DQ genotype				
-DR4-DQ8/DR4-DQ8	230 (19.0)	2235 (42.1)	73 (16.0)	2392 (39.5)
-DR3-DQ2/DR4-DQ8	477 (39.4)	2176 (41.0)	153 (33.7)	2500 (41.2)
-DR3-DQ2/DR3-DQ2	505 (41.6)	897 (16.9)	229 (50.3)	1173 (19.3)
Birth year				
2004–5	217 (17.9)	856 (16.1)	105 (23.0)	968 (16.0)
2006	243 (20.0)	887 (16.7)	76 (16.7)	1054 (17.4)
2007	244 (20.1)	1141 (21.5)	90 (19.8)	1295 (21.3)
2008	220 (18.2)	1104 (20.8)	80 (17.6)	1244 (20.5)
2009–10	288 (23.8)	1320 (24.9)	104 (22.9)	1504 (24.8)
Mode of delivery—Cesarean section				
Yes	262 (21.6)	1394 (26.3)	85 (18.7)	1571 (25.9)
No	950 (78.4)	3910 (73.7)	370 (81.3)	4490 (74.1)
Mother’s education				
—more than high school	1016 (85.2)	4203 (81.5)	368 (82.5)	4851 (82.1)
Duration of exclusive breastfeeding at least 3 months	363 (30.0)	1275 (24.0)	143 (31.4)	1495 (24.7)
Exposure to probiotics by the age of 12 months	281 (23.2)	1179 (22.2)	99 (21.8)	1361 (22.4)
Source of first exposure to probiotics by the age of 12 months				
-Dietary supplement	238 (19.6)	949 (17.9)	83 (18.3)	1104 (18.2)
-Infant formula	42 (3.5)	228 (4.3)	16 (3.5)	254 (4.2)
-Both	1 (0.1)	2 (0.0)	0 (0.0)	3 (0.0)
Age (weeks) at first probiotic exposure among probiotics users				
median (Q1–Q3)	5 (2, 26)	6 (2, 19)	5 (3, 26)	6 (2, 20)
mean (SD)	13.4 (14.7)	12.1 (13.2)	14.5 (15.3)	12.2 (13.3)
Age (weeks) at first probiotic exposure among probiotics users with first exposure from dietary supplements				
median (Q1–Q3)	4 (2, 20)	5 (3, 17)	4 (2, 26)	5 (2, 17)
mean (SD)	12.5 (14.5)	11.8 (13.3)	13.6 (15.4)	11.8 (13.4)
Age (weeks) at first probiotic exposure among probiotics users with first exposure from infant formula				
median (Q1–Q3)	20 (4, 28)	9 (2, 24)	22 (4, 26)	10 (2, 24)
mean (SD)	19.0 (14.9)	13.4 (12.6)	19.0 (14.4)	14.0 (13.0)

Data are presented as number (percentage) unless otherwise indicated.

**Table 2:** Characteristics of probiotics users by source of first exposure and non-users during the first year of life.

	Source of First Probiotic Exposure among Probiotics Users during the First Year of Life ^a^		Non-Users of Probiotics (*n* = 5060)	*p* ^b^	*p* ^c^
	Dietary Supplements (*n* = 1187)	Infant Formula (*n* = 270)			
Country				<0.001	<0.001
US	119 (10.0)	49 (18.1)	2464 (48.7)		
Finland	776 (65.4)	29 (10.7)	678 (13.4)		
Germany	10 (0.8)	154 (57.0)	182 (3.6)		
Sweden	282 (23.8)	38 (14.1)	1736 (34.3)		
Family member with celiac disease				0.736	0.600
Yes	62 (5.2)	8 (3.0)	189 (3.7)		
No	1125 (94.8)	262 (97.0)	4871 (96.3)		
Sex				0.814	0.472
Male	614 (51.7)	133 (49.3)	2584 (51.1)		
Female	573 (48.3)	137 (50.7)	2476 (48.9)		
HLA DR-DQ genotype				0.533	0.061
-DR4-DQ8/DR4-DQ8	519 (43.7)	83 (30.7)	1862 (36.8)		
-DR3-DQ2/DR4-DQ8	446 (37.6)	137 (50.7)	2069 (40.9)		
-DR3-DQ2/DR3-DQ2	222 (18.7)	50 (18.5)	1129 (22.3)		
Birth year				<0.001	<0.001
2004–5	97 (8.2)	45 (16.7)	930 (18.4)		
2006	168 (14.2)	38 (14.1)	924 (18.3)		
2007	260 (21.9)	47 (17.4)	1077 (21.3)		
2008	272 (22.9)	64 (23.7)	988 (19.5)		
2009–10	390 (32.9)	76 (28.1)	1141 (22.5)		
Mode of delivery: Cesarean section				0.012	0.281
Yes	238 (20.1)	99 (36.7)	1319 (26.1)		
No	949 (79.9)	171 (63.3)	3737 (73.9)		
Birth order, first child				<0.001	0.314
Yes	575 (50.0)	131 (50.0)	2127 (43.4)		
No	575 (50.0)	131 (50.0)	2778 (56.6)		
Mother’s age at delivery (years)				0.001	0.177
≤24	107 (9.0)	18 (6.7)	604 (11.9)		
25–29	402 (33.9)	73 (27.0)	1437 (28.4)		
30–34	424 (35.7)	113 (41.9)	1798 (35.5)		
>34	254 (21.4)	66 (24.4)	1221 (24.1)		
Mother’s education				<0.001	0.133
High school or less	123 (10.6)	36 (13.8)	974 (19.8)		
More than high school	1038 (89.4)	225 (86.2)	3954 (80.2)		
Maternal pre-pregnancy body mass index				0.462	0.278
≤25	776 (66.6)	180 (67.2)	3057 (61.6)		
>25	390 (33.4)	88 (32.8)	1905 (38.4)		
Smoking during pregnancy				0.005	0.334
Yes	119 (10.2)	34 (12.6)	552 (11.0)		
No	1052 (89.8)	236 (87.4)	4460 (89.0)		
Maternal antibiotic use during pregnancy				0.363	0.605
Yes	289 (24.7)	55 (20.4)	1122 (22.4)		
No	882 (75.3)	215 (79.6)	3896 (77.6)		
Maternal probiotics use during pregnancy				<0.001	0.317
Yes	91 (7.7)	13 (4.8)	148 (2.9)		
No	1096 (92.3)	257 (95.2)	4912 (97.1)		
Duration of exclusive breastfeeding				<0.001	<0.001
<3 months or none	887 (74.8)	225 (83.3)	3766 (74.4)		
≥3 months	299 (25.2)	45 (16.7)	1293 (25.6)		
Age at gluten introduction				0.313	0.027
<17 weeks	69 (5.9)	11 (4.2)	312 (6.3)		
17–26 weeks	425 (36.1)	76 (28.7)	1818 (36.5)		
>26 weeks	684 (58.1)	178 (67.2)	2851 (57.2)		
Child antibiotic use during the first 12 months				<0.001	0.849
Yes	728 (61.3)	125 (46.3)	2259 (44.6)		
No	459 (38.7)	145 (53.7)	2801 (55.4)		
Diarrhea during the first 3 months				<0.001	0.268
Yes	115 (9.7)	44 (16.3)	419 (8.3)		
No	1072 (90.3)	226 (83.7)	4641 (91.7)		
Gastrointestinal infections during the first 12 months				<0.001	0.019
Yes	415 (35.0)	92 (34.1)	1496 (29.6)		
No	772 (65.0)	178 (65.9)	3564 (70.4)		
Common cold during the first 3 months				0.006	0.055
Yes	677 (57.0)	142 (52.6)	2955 (58.4)		
No	510 (43.0)	128 (47.4)	2103 (41.6)		
Age at first exposure to probiotics (weeks)					0.005
Mean (SD)	12 (14)	14 (13)			
Median (IQR)	5 (2–17)	10 (2–24)			
Duration of probiotic exposure during the first year of life (weeks)					0.073
Mean (SD)	30 (18)	25 (18)			
Median (IQR)	35 (11–48)	23 (8–44)			

^a^: Three children were exposed to both probiotic dietary supplements and infant formula at the same time and were not included here. ^b^: *p* value from the Cochran–Mantel–Haenszel test for the association of characteristics between probiotics users and non-users during first year of life; analyses adjusted for country. ^c^: *p* value from the Cochran–Mantel–Haenszel test (on categorical variables) or the analysis of covariance (on continuous variables) for the association of characteristics between the sources of first probiotic exposure; analyses adjusted for country. Data are presented as number (percentage) unless otherwise indicated.

**Table 3:** Overall probiotic exposure, timing of first probiotic exposure by source, and the risk of celiac disease auto-immunity (CDA) and celiac disease.

	CDA	Celiac Disease		
	HR (95% CI) ^a^ *p*	HR (95% CI) ^b^ *p*	HR (95% CI) ^a^ *p*	HR (95% CI) ^b^ *p*
Exposed to probiotics during the first year of life:				
Yes vs. no	1.11 (0.96, 1.29) 0.177	1.15 (0.99, 1.35) 0.073	1.04 (0.82, 1.33) 0.731	1.11 (0.86, 1.43) 0.432
First exposure to probiotics by source:				
Supplements vs. none	1.15 (0.98, 1.35) 0.085	1.18 (1.01, 1.40) 0.043	1.03 (0.79, 1.35) 0.804	1.09 (0.83, 1.44) 0.534
Formula vs. none	0.91 (0.64, 1.30) 0.616	0.98 (0.69, 1.41) 0.930	1.12 (0.63, 1.98) 0.695	1.20 (0.68, 2.13) 0.537
Age at first exposure to probiotics among users (/week) ^c,d^	1.01 (1.00, 1.02) 0.047	1.01 (1.00, 1.02) 0.210	1.02 (1.01, 1.04) 0.009	1.02 (1.00, 1.03) 0.055
Age at first exposure to probiotics among users whose first exposure to probiotics were from dietary supplements (/week) ^c^	1.00 (0.99, 1.01) 0.478	1.00 (0.99, 1.01) 0.618	1.01 (1.00, 1.03) 0.102	1.01 (0.99, 1.03) 0.218
Age at first exposure to probiotics among users whose first exposure to probiotics were from infant formula (/week) ^c^	1.03 (1.01,1.05) 0.014	1.02 (0.99, 1.05) 0.285	1.04 (1.00, 1.09) 0.044	1.04 (0.99, 1.09) 0.135

^a^ Hazard ratios adjusted for sex, HLA genotype, first-degree relative (FDR) with celiac disease and country. ^b^ Hazard ratios adjusted for sex, HLA genotype, FDR with celiac disease, country, birth year, mode of delivery, mother’s education, duration of exclusive breastfeeding, and child’s diarrhea during first 3 months. (Birth year, mode of delivery, mother’s education, duration of exclusive breastfeeding, and child’s diarrhea during first 3 months were statistically significantly (*p*-value < 0.05) associated with probiotic exposure during the first year of life, and with celiac disease autoimmunity (CDA) and/or celiac disease.) ^c^ Hazard ratios describe the change in the risk for every one week delay in the probiotic exposure. ^d^ Hazard ratios adjusted additionally for the source of probiotics.

## Supplementary Materials

The following are available online at https://www.mdpi.com/2072-6643/11/8/1790/s1, Figure S1: Flowchart describing the study population, Figure S2: Probiotic supplementation (%) during the first year of life by year of birth and by country, Table S1: High risk HLA genotypes followed in TEDDY.

## References

[1] Food and Agriculture Organization of the United Nations, World Health Organization (2006). Probiotics in Food: Health and Nutritional Properties and Guidelines for Evaluation. http://www.fao.org/3/a-a0512e.pdf.

[2] Sanders M.E., Heimbach J.T., Pot B., Tancredi D.J., Lenoir-Wijnkoop I., Lähteenmäki-Uutela A., Gueimonde M., Banares S. (2010). Safety assessment of probiotics for human use. Gut Microbes.

[3] Bergmann H., Rodriquez J.M., Salminen S., Szajewska H. (2014). Probiotics in human milk and probiotic supplementation in infant nutrition: A workshop report. Br. J. Nutr..

[4] Martin R., Langa S., Reviriego C., Jiminez E., Marin M.L., Xaus J., Fernández L., Rodríguez J.M. (2003). Human milk is a source of lactic acid bacteria for the infant gut. J. Pediatr..

[5] Allen S.J., Jordan S., Storey M., Thornton C.A., Gravenor M.B., Garaiova I., Plummer S.F., Wang D., Morgan G. (2014). Probiotics in the prevention of eczema: A randomized controlled trial. Arch. Dis. Child..

[6] Zuccotti G., Meneghin F., Aceti A., Barone G., Callegari M.L., Di Mauro A., Fantini M.P., Gori D., Indrio F., Maggio L. (2015). Probiotics for prevention of atopic diseases in inafnts: Systematic review and meta-analysis. Allergy.

[7] Perceval C., Szajewska H., Indrio F., Weizman Z., Vandenplas Y. (2019). Profylactic use of probiotics for gastrointestinal disorders in children. Lancet Child Adolesc. Health.

[8] Indrio F., Di Mauro A., Riezzo G., Civardi E., Intini C., Corvaglia L., Ballardini E., Bisceglia M., Brazzoduro E., Del Vecchio A. (2014). Prophylactic use of a probiotic in the prevention of colic, regurgitation, and functional constipation: A randomized clinical trial. JAMA Pediatr..

[9] Anabrees J., Indrio F., Paes B., AlFaleh K. (2013). Probiotics for infantile colic: A systematic review. BMC Pediatr..

[10] Sung V., Hiscock H., Tang M.L., Mensah F.K., Nation M.L., Satzke C., Heine R.G., Stock A., Barr R.G., Wake M. (2014). Treating infant colic with probiotic *Lactobacillus reuteri*: Double blind, placebo controlled randomized trial. BMJ.

[11] Savino F., Garro M., Montanari P., Galliano I., Bergallo M. (2018). Crying Time and RORγ/FOXP3 Expression in *Lactobacillus reuteri* DSM17938-Treated Infants with Colic: A Randomized Trial. J. Pediatr..

[12] Uusitalo U., Liu X., Yang J., Aronsson A.C., Hummel S., Butterworth M., Lernmark Å., Rewers M., Hagopian W., She J.X. (2016). Association of early exposure of probiotics and islet autoimmunity in the TEDDY Study. JAMA Pediatr..

[13] Yang J., Tamura R., Uusitalo U., Aronsson C.A., Silvis K., Riikonen A., Frank N., Joslowski G., Winkler C., Norris J.M. (2017). Vitamin D and probiotics supplement use in young children with genetic risk for type 1 diabetes. Eur. J. Clin. Nutr..

[14] Salminen M.K., Tynkkynen S., Rautelin H., Saxelin M., Vaara M., Ruutu P., Sarna S., Valtonen V., Järvinen A. (2002). Lactobacillus bacteremia during a rapid increase in probiotic use of *Lactobacillus rhamnosus* GG in Finland. Clin. Infect. Dis..

[15] Plaza-Diaz J., Ruiz-Ojeda F.J., Gil-Campos M., Gil A. (2019). Mechanisms of action of probiotics. Adv. Nutr..

[16] Sollid L.M. (2000). Molecular basis of celiac disease. Annu. Rev. Immunol..

[17] Aronsson A.C., Lee H.S., Koletzko S., Uusitalo U., Yang J., Virtanen S.M., Liu E., Lernmark Å., Norris J.M., Agardh D. (2016). Effects of gluten intake on risk of celiac disease: A case-control study on a Swedish birth cohort. Clin. Gastroenterol. Hepatol..

[18] Plot L., Amital H. (2009). Infectious associations of celiac disease. Autoimmun. Rev..

[19] Mårild K., Kahrs C.R., Tapia G., Stene L.C., Størdal K. (2015). Infections and risk of celiac disease in childhood: A prospective nationwide cohort study. Am. J. Gastroenterol..

[20] Kemppainen K.M., Vehik K., Lynch K.F., Larsson H.E., Canepa R.J., Simell V., Koletzko S., Liu E., Simell O.G., Toppari J. (2017). Association between early-life antibiotic use and the risk of islet or celiac disease autoimmunity. JAMA Pediatr..

[21] Pozo-Rubio T., Olivares M., Nova E., De Palma G., Mujico J.R., Ferrer M.D., Marcos A., Sanz Y. (2012). Immune development and intestinal microbiota in celiac disease. Clin. Dev. Immunol..

[22] De Sousa Moraes L.F., Grzeskowiak L.M., de Sales Teixeira T.F., Peluzio M.D.C.G. (2014). Intestinal microbiota and probiotics in celiac disease. Clin. Microbiol. Rev..

[23] Olivares M., Casteillejo G., Varea V., Sanz Y. (2014). Double-blind, randomized, placebo-controlled intervention trial to evaluate the effects of *Bifidobacterium longum* CECT 7347 in children with newly diagnosed coeliac disease. Br. J. Nutr..

[24] Klemenak M., Dolinšek J., Langerholc T., Di Gioia D., Mičetić-Turk D. (2015). Administration of *Bifidobacterium breve* decreases the production of TNF-α in children with celiac disease. Dig. Dis. Sci..

[25] Lernmark B., Johnson S.B., Vehik K., Smith L., Ballard L., Baxter J., McLeod W., Roth R., Simell T. (2011). Enrollment experiences in a pediatric longitudinal observation study: The Environmental Determinants of Diabetes in the Young (TEDDY) Study. Contemp. Clin. Trials.

[26] Vehik K., Fiske S.W., Logan C.A., Agardh D., Cilio C.M., Hagopian W., Simell O., Roivainen M., She J.X., Briese T. (2013). Methods, Quality Control and Specimen Management in an International Multi-Center Investigation of Type 1 Diabetes: TEDDY. Diabetes Metab. Res. Rev..

[27] Agardh D., Lee H.S., Kurppa K., Simell V., Aronsson C.A., Jörneus O., Hummel M., Liu E., Koletzko S., TEDDY Study Group (2015). Clinical features of celiac disease: A preospective birth cohort. Pediatrics.

[28] Therneau T.M., Grambsch P.M. (2000). Modeling Survival Data: Extending the Cox Model.

[29] Therneau T. (2015). A Package for Survival Analysis in S. Version 2.38 in R 3.0.

[30] Håkansson A., Andren Aronsson C., Brundin C., Oscarsson E., Molin G., Agardh D. Lactobacillus plantarum HEAL9 and Lactobacillus paracasei 8700:2 suppress ongoing celiac autoimmunity in children at genetic risk for developing celiac disease. Proceedings of the 17th International Celiac Disease Symposium.

[31] Aronsson C.A., Lee H.S., Liu E., Uusitalo U., Hummel S., Yang J., Hummel M., Rewers M., She J.X., Simell O. (2015). Age at gluten introduction and risk of celiac disease. Pediatrics.

[32] Pinto-Sánchez M.I., Verdu E.F., Liu E., Bercik P., Green P.H., Murray J.A., Guandalini S., Moayyedi P. (2016). Gluten introduction to infant feeding and risk of celiac disease: Systematic review and meta-analysis. J. Pediatr..

[33] Akobeng A.K., Ramanan A.V., Buchan I., Heller R.F. (2006). Effect of breast feeding on risk of coeliac disease: A systematic review and meta-analysis of observational studies. Arch. Dis. Child..

[34] Bittker S.S., Bell K.R. (2019). Potential risk factors for celiac disease in childhood: A case-control epidemiological survey. Clin. Exp. Gastroenterol..

[35] Sander S.D., Andersen A.M.N., Murray J.A., Karlstad Ø., Husby S., Størdal K. (2019). Association between antibiotics in the first year of life and celiac disease. Gastroenterology.

[36] Chau K., Lau E., Greenberg S., Jacobson S., Yazdani-Brojeni P., Verma N., Koren G. (2015). Probiotics for infantile colic: A randomized, double-blind, placebo-controlled trial investigating *Lactobacillus reuteri* DSM 17938. J. Pediatr..

[37] Pärtty A., Isolauri E. (2012). Gut microbiota and infant distress—The association between compositional development of the gut microbiota and fussing and crying in early infancy. Microb. Ecol. Health Dis..

[38] De Weerth C., Fuentes S., Puylaert P., de Vos W.M. (2013). Intestinal microbiota of infants with colic: Development and specific signatures. Pediatrics.

[39] Cenit M.C., Olivares M., Codoner-Franch P., Sanz Y. (2015). Intestinal microbiota and celiac disease: Cause, consequence or co-evolution?. Nutrients.

[40] Gelfand A.A., Thomas K.C., Goadsby P.J. (2012). Before the headache. Infant colic as an early life expression of migraine. Neurology.

[41] Van Hemert S., Breedveld A.C., Rovers J.M., Vermeiden J.P., Witteman B.J., Smits M.G., de Roos N.M. (2014). Migraine associated with gastrointestinal disorders: Review of the literature and clinical implications. Front. Neurol..

[42] Laursen M.F., Bahl M.I., Michaelsen K.F., Licht T.R. (2017). First foods and gut microbes. Front. Microbiol..

[43] Cristofori F., Indrio F., Miniello V.L., De Angelis M., Francavilla R. (2018). Probiotics in celiac disease. Nutrients.

[44] Gueimonde M., Sánchez B. (2012). Enhancing probiotic stability in industrial processes. Microb. Ecol. Health Dis..

