# Supplementary material for: Early Probiotic Supplementation and the Risk of Celiac Disease in Children at Genetic Risk
Source: Nutrients. 2019 Aug 2;11(8):1790. doi: 10.3390/nu11081790 (PMC6722940; doi:10.3390/nu11081790)
Supplement: Supplementary file 1 [file nutrients-11-01790-s001.zip › nutrients-543767-suppl/Prob_CD_Supplemental Table 1.docx]

**Table S1**. High risk HLA genotypes followed in TEDDY.

| **HLA genotypes** | | **Abbreviation** | **FDR with T1D** | **General population** |
| --- | --- | --- | --- | --- |
| **A** | **DR4-DQA1*03:0X-DQB1*03:02 / DR3-DQA1*05:01-DQB1*02:01** | **DR3-DQ2/DR4-DQ8** | **Y** | **Y** |
| **B** | **DR4-DQA1*03:0X-DQB1*03:02 / DR4-DQA1*03:0X-DQB1*03:02** | **DR4-DQ8/DR4-DQ8** | **Y** | **Y** |
| **C** | **DR4-DQA1*03:0X-DQB1*03:02 / DR8-DQA1*04:01-DQB1*04:02** | **DR4-DQ8/DR8-DQ4** | **Y** | **Y** |
| **D** | **DR3-DQA1*05:01-DQB1*02:01 / DR3-DQA1*05:01-DQB1*02:01** | **DR3-DQ2/DR3-DQ2** | **Y** | **Y** |
| E | DR4-DQA1*03:0X-DQB1*03:02 / DR4- DQA1*03:0X-DQB1*02:0X | DR4/DR4b | Y | N |
| F | DR4-DQA1*03:0X-DQB1*03:02 / DR1- DQA1*01:01-DQB1*05:01 | DR4/DR1 | Y | N |
| G | DR4-DQA1*03:0X-DQB1*03:02 / DR13-DQA1*01:02-DQB1*06:04 | DR4/DR13 | Y | N |
| H | DR4-DQA1*03:0X-DQB1*03:02 / DR9- DQA1*03:0X-DQB1*03:03 | DR4/DR9 | Y | N |
| I | DR3-DQA1*05:01-DQB1*02:01 / DR9- DQA1*030:X-DQB1*03:03 | DR3/DR9 | Y | N |

Abbreviations: FDR with T1D; first degree relative with type 1 diabetes

The genotypes reported in this study are in bold.

Note: For general population subjects, DR4 subtyping must exclude DRB1*04:03. Acceptable alleles in the DR4 haplotype include both DQB1*03:02 (shown) and *03:04 (not shown). DR8-DQA1*04:01-DQB1*04:02 is the only low risk HLA haplotype for celiac disease present in greater than 2% of subjects in the study cohort.
