# Supplementary figures and images for: Early Probiotic Supplementation and the Risk of Celiac Disease in Children at Genetic Risk
Source: Nutrients. 2019 Aug 2;11(8):1790. doi: 10.3390/nu11081790 (PMC6722940; doi:10.3390/nu11081790)

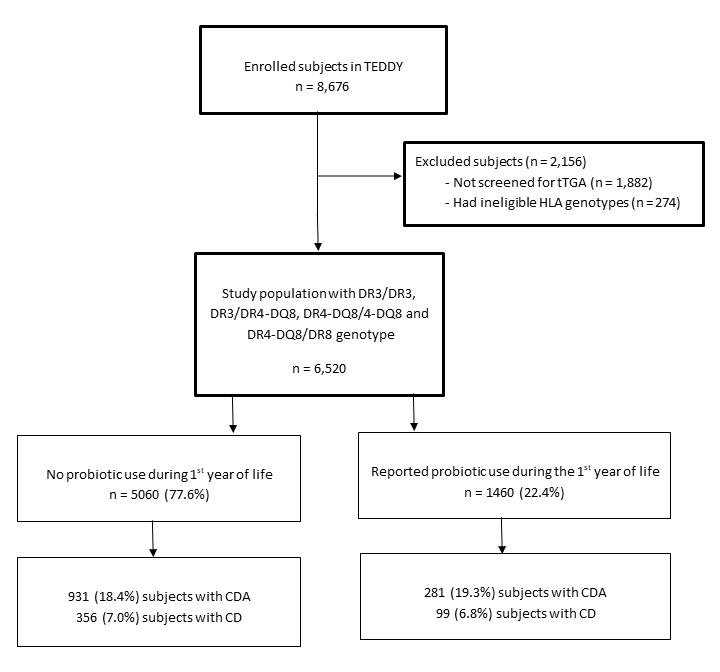

Supplement: Supplementary file 1 [file nutrients-11-01790-s001.zip › nutrients-543767-suppl/SupplementalFigure1.tif]

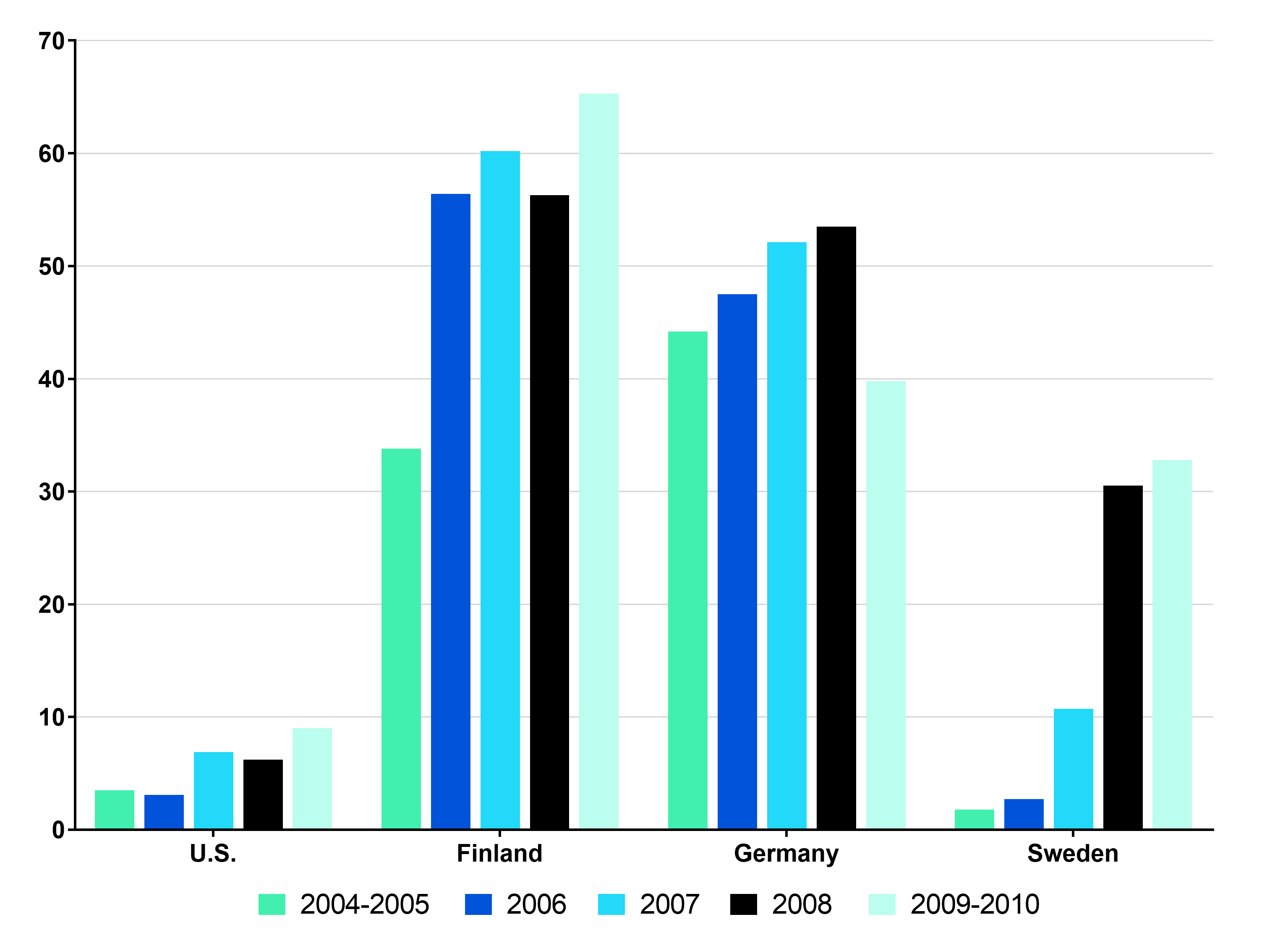

Supplement: Supplementary file 1 [file nutrients-11-01790-s001.zip › nutrients-543767-suppl/SupplementalFigure2.tif]
